# The effects of antioxidant supplementation on pain, oxidative stress markers, and clinical pregnancy rate in women with endometriosis: a systematic review and meta-analysis of randomized controlled trials

**DOI:** 10.3389/fmed.2025.1694281

**Published:** 2025-10-29

**Authors:** Yuchan Zhong, Xinyu Qiao, Xin Huang, Yujing Li, Ruiying Wang, Jiagui Liang, Tingting Liu, Wenjie Bo, Huiqiao Lai, Wei Huang

**Affiliations:** ^1^Division of Reproductive Medicine, West China Second University Hospital of Sichuan University, Chengdu, Sichuan, China; ^2^Key Laboratory of Birth Defects and Related Diseases of Women and Children of Ministry of Education, Chengdu, Sichuan, China; ^3^NHC Key Laboratory of Chronobiology (Sichuan University), Chengdu, Sichuan, China

**Keywords:** endometriosis, antioxidant, pelvic pain, dysmenorrhea, malondialdehyde, clinical pregnancy rate

## Abstract

Endometriosis is a chronic inflammatory disease characterized by pelvic pain and infertility, with oxidative stress playing a key role in its pathogenesis. Although antioxidant supplementation has been proposed as a potential adjunctive therapy in endometriosis, current evidence regarding its efficacy in symptom relief and fertility improvement remains inconclusive. This systematic review and meta-analysis included 21 randomized controlled trials involving 1,626 participants and evaluated more than 10 types of antioxidant supplementation, including vitamins, pentoxifylline, melatonin, astaxanthin, fish oil, and silymarin. The results showed that antioxidant supplementation significantly alleviated pelvic pain (continuous outcomes: SMD = −2.68; *p* < 0.00001; binary outcomes: RR = 9.31; *p* < 0.0001), dysmenorrhea (SMD = −1.77; *p* = 0.01; RR = 2.39; *p* = 0.03), and dyspareunia (SMD = −2.33; *p* = 0.01; RR = 5.40; *p* = 0.003), and significantly decreased peripheral blood malondialdehyde (MDA) levels (SMD = −7.58; *p* = 0.001). However, no significant effects were observed on overall pain (SMD = −1.14; *p* = 0.51) or clinical pregnancy rate (RR = 1.12; *p* = 0.52). Subgroup analyses further indicated that treatment efficacy varied by antioxidant type, disease stage, and duration of intervention. These findings suggest that antioxidant supplementation may offer therapeutic benefits in alleviating specific pain symptoms and reducing oxidative stress in women with endometriosis. Further large-scale and high-quality randomized controlled trials are needed to validate these results and establish optimal antioxidant strategies for long-term management of endometriosis.

**Systematic review registration:**

PROSPERO CRD420251071723, https://www.crd.york.ac.uk/prospero/display_record.php?ID=CRD420251071723.

## 1 Introduction

Endometriosis is defined as the presence of endometrial-like tissue outside the uterine cavity, with an estimated prevalence of up to 10% among women of reproductive age (1). As a chronic systemic condition, endometriosis is associated with symptoms such as pelvic pain and infertility, significantly impairing patients’ quality of life (2). The exact etiology of endometriosis remains unclear, with current predominant theories including retrograde menstruation, coelomic metaplasia, and Müllerian remnant abnormalities (3). In recent years, oxidative stress has been increasingly recognized as a key contributor to the pathogenesis and progression of endometriosis (4, 5). It refers to an imbalance between oxidants and antioxidants, where excessive oxidants disrupt redox signaling and cause molecular damage (6). Numerous studies have demonstrated elevated reactive oxygen species (ROS) in the peritoneal fluid and peripheral blood of women with endometriosis, accompanied by reduced antioxidant enzyme activity and accumulation of lipid peroxidation products (7). This imbalance may promote lesion formation and pain via enhanced adhesion, invasion, and immune evasion of ectopic endometrial cells (4, 8, 9). Oxidative stress also contributes to oocyte aging, impair ovarian function, and reduces embryo implantation, ultimately reducing fertility in individuals with endometriosis (10, 11).

Currently, first-line treatment options for endometriosis include surgical interventions and pharmacological therapies such as hormonal agents and nonsteroidal anti-inflammatory drugs (NSAIDs) (12, 13). However, these approaches are often associated with limited efficacy, notable side effects, or high recurrence rates, highlighting the urgent need for safer and more sustainable adjunctive therapies (12, 13). Antioxidants, also known as free radical scavengers, are compounds capable of inhibiting oxidative processes by neutralizing free radicals (14). They are broadly classified into exogenous antioxidants and endogenous antioxidant systems including enzymatic and non-enzymatic components (15). Antioxidants have been reported to reduce the incidence of various diseases such as cancer, diabetes, inflammation, and cardiovascular disorders (14, 16). Given their favorable safety profile and potential multitarget effects, antioxidants have been proposed as promising adjunctive agents in endometriosis management. Preliminary clinical studies have indicated that certain antioxidants, such as combined vitamins C and E, melatonin, and pentoxifylline, may alleviate pain, modulate oxidative stress markers, and even improve pregnancy outcomes in endometriosis patients (17–19). Nevertheless, other studies did not demonstrate significant clinical benefits (20–22). The existing evidence is limited by small sample sizes and methodological heterogeneity. Previous systematic reviews have mainly focused on individual antioxidants (e.g., vitamin D, melatonin) or single outcomes (e.g., pain, pregnancy), limiting the generalizability of findings (23–25). Therefore, a comprehensive and systematic evaluation that simultaneously assesses the overall efficacy of multiple antioxidant supplements across several key clinical outcomes is still lacking.

This study aims to address this gap by conducting a systematic review and meta-analysis of randomized controlled trials (RCTs) to synthesize current evidence on the efficacy of different antioxidants in improving pain, modulating oxidative stress levels, and enhancing clinical pregnancy rate in women with endometriosis. Furthermore, we investigated potential influencing factors such as the type of antioxidant, duration of intervention, and disease stage, with the aim of providing higher-quality evidence and practical guidance for clinical decision-making.

## 2 Methods

This systematic review was conducted in accordance with the Preferred Reporting Items for Systematic Reviews and Meta-Analyses (PRISMA) guidelines (26) and has been registered in the International Prospective Register of Systematic Reviews (PROSPERO) under the number CRD420251071723.^1^

### 2.1 Search strategy

Two reviewers independently searched the PubMed, Embase, Web of Science, and Cochrane Central Register of Controlled Trials (CENTRAL) databases from inception to May 14, 2025. The search terms included:

(“Antioxidants” OR “Anti-Oxidant” OR “Anti Oxidant” OR “Anti-Oxidants” OR “Anti Oxidants” OR “Antioxidant” OR “Antioxidant Effect” OR “Antioxidant Effects” OR “Anti-Oxidant Effect” OR “Anti Oxidant Effect” OR “Anti-Oxidant Effects” OR “Anti Oxidant Effects” OR “Antioxidant Activity” OR “Activity, Antioxidant” OR “Endogenous Antioxidants” OR “Antioxidants, Endogenous” OR “Endogenous Antioxidant” OR “Antioxidant, Endogenous”) AND (“Endometriosis” OR “Endometrioses” OR “Endometrioma” OR “Endometriomas”). In addition, the reference lists of relevant systematic reviews identified through the database search were manually screened to ensure a comprehensive search.

### 2.2 Eligibility criteria

The inclusion criteria were established based on the PICOS strategy, as shown in Table 1.

**Table 1:** PICOS strategy of the study.

PICOS strategy of the study		
P (participants/patients)	Women diagnosed with endometriosis by laparoscopy, histopathology, or imaging	
I (intervention)	One or more antioxidant supplements	Both intervention and control groups may concurrently receive standard first-line treatments for endometriosis (hormonal therapy or analgesics)
C (comparison)	Placebo or no treatment	
O (outcomes)	**Primary outcome:** Pain symptoms, assessed by VAS, including: Pelvic pain: pain localized to the pelvic region Dysmenorrhea: pain during menstruation Dyspareunia: pain during intercourse Overall pain: a general category of pain not specific to any particular location or type, including pain described as “worst pain,” “endometriosis-associated pain,” or simply “pain.” **Secondary outcomes:** Oxidative stress biomarkers, including: MDA, TAC, SOD, and LOOH Clinical pregnancy rate **Measurement timepoints:** All outcomes were assessed at baseline (pre-intervention) and at the end of the intervention	
S (study design)	RCTs only	

VAS, Visual Analog Scale; MDA, Malondialdehyde; TAC, Total Antioxidant Capacity; SOD, Superoxide Dismutase; LOOH, Lipid Hydroperoxide; RCTs, Randomized Controlled Trials.

The exclusion criteria were as follows: (1) non-randomized designs, including cohort studies, case–control studies, quasi-randomized trials, case reports, reviews, meta-analyses, *in vitro* or animal studies; (2) studies with imbalanced standard endometriosis treatments between groups or those in which standard treatment might significantly affect outcomes; (3) studies without full text, lacking relevant outcome data, or with incomplete results; (4) studies not published in English.

Two reviewers independently conducted study selection, including an initial screening based on titles and abstracts, followed by full-text review. Disagreements during the selection process were resolved through discussion with a third reviewer.

### 2.3 Data extraction

Two reviewers independently extracted data from the included studies. Extracted data included: first author’s name, year of publication, country, sample size, participant characteristics (age, diagnostic method, stage of endometriosis), intervention details (type and dosage of antioxidant, duration of intervention, presence or absence of placebo, concurrent use of standard endometriosis treatments), and outcomes (pain, oxidative stress biomarkers, clinical pregnancy rate).

Additionally, for the study by Nodler et al. (ID: 7) which included both Vitamin D3 and fish oil intervention groups, data from different intervention arms were treated as independent comparisons. To avoid statistical bias from control group duplication, the control group sample size was halved for each comparison. However, risk of bias assessment was still performed at the original study level.

### 2.4 Risk of bias

Two reviewers independently assessed the risk of bias of the included studies. Any disagreements were resolved through discussion with a third reviewer. Risk of bias was evaluated using the latest version of Cochrane Risk of Bias tool (RoB2), which includes five domains: randomization process, deviations from the intended interventions, missing outcome data, measurement of the outcome, and selection of the reported result. Each domain was judged as “low risk,” “some concerns,” or “high risk” (27).

### 2.5 Evidence quality assessment

Two reviewers independently assessed the quality of evidence for each outcome using the online GRADEpro GDT system.^2^ The certainty of evidence was rated as “high,” “moderate,” “low,” or “very low.” Since only RCTs were included in this study, the initial level of certainty for all outcomes started as “high.” The certainty of evidence could be downgraded for five reasons: risk of bias, inconsistency of results, indirectness of evidence, imprecision, and publication bias. Conversely, three factors could lead to upgrading the certainty: large magnitude of effect, plausible residual confounding, and dose–response gradient.

### 2.6 Statistical analysis

Meta-analyses were performed using Review Manager (RevMan) version 5.4. For outcomes with data available from at least three studies, quantitative synthesis was conducted. For continuous variables, the mean and standard deviation (SD) before and after intervention in both groups were obtained or calculated, and the SD of the change was computed using the following formula:

SD(change) = √[SD(pre)^2^ + SD(post)^2^–2 × R × SD(pre) × SD(post)], where R = 0.5 (27). Heterogeneity was assessed using Cochran’s Q test and quantified using the I^2^ statistic. A fixed-effect model was applied if *I*^2^ < 50%, while a random-effects model was used if *I*^2^ ≥ 50%. The pooled effect size for continuous outcomes was reported as mean difference (MD) with 95% confidence intervals (CI); for dichotomous outcomes, odds ratios (OR) with 95% CI were reported. A *p*-value < 0.05 was considered statistically significant. For outcomes with substantial heterogeneity, sensitivity analyses (leave-one-out approach) and subgroup analyses (e.g., type of antioxidant, concurrent hormonal therapy) were conducted to explore potential sources of heterogeneity. Funnel plots were not used to assess publication bias due to the inclusion of fewer than 10 studies for each outcome.

## 3 Results

### 3.1 Search results

**Figure 1:**
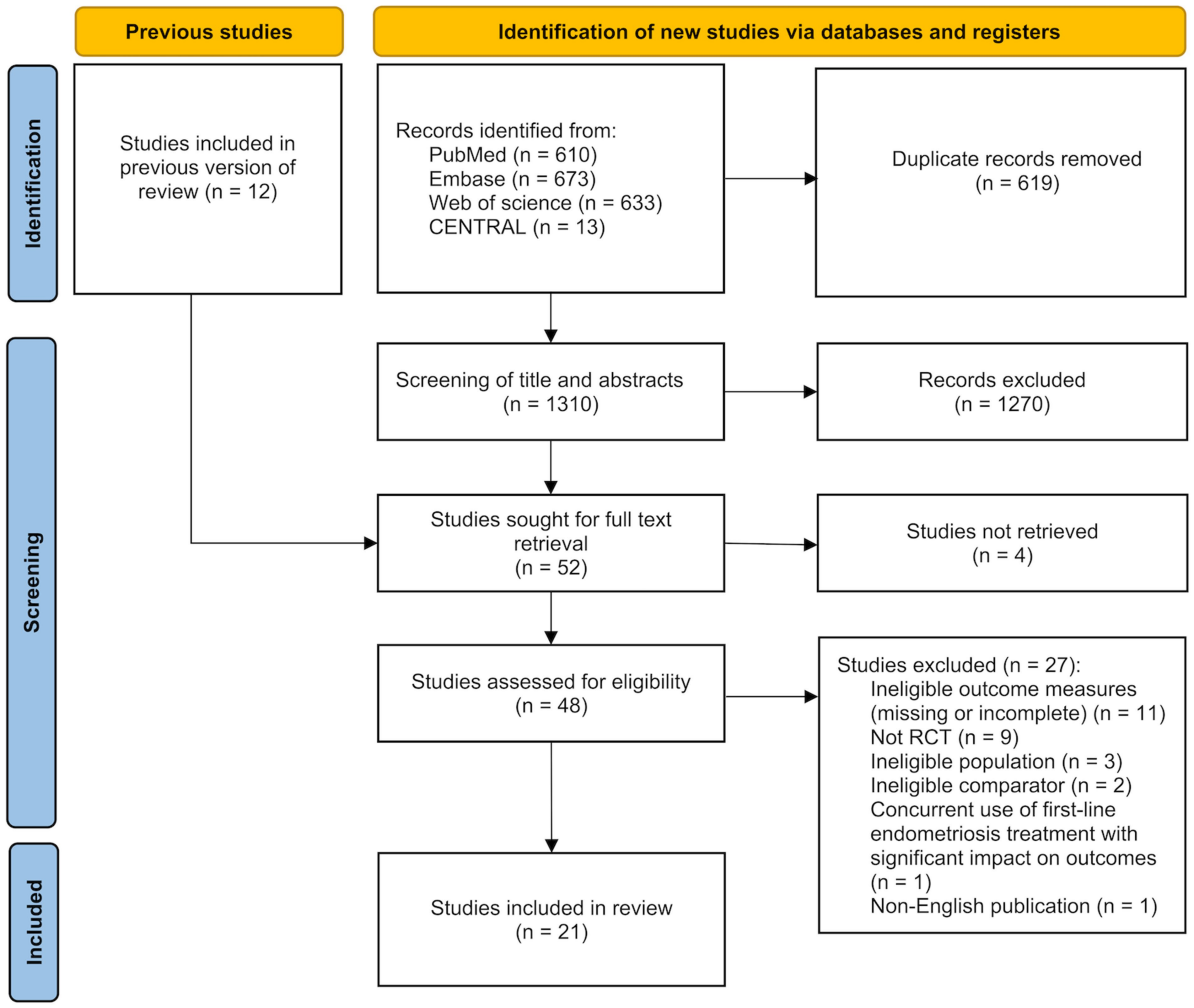
PRISMA flow diagram of included studies.

The study selection process is illustrated in Figure 1. A comprehensive search of PubMed, Embase, Web of Science, and the Cochrane Central Register of Controlled Trials (CENTRAL) was conducted on May 14, 2025 and yielded a total of 1,929 records. After removing 619 duplicates, the titles and abstracts of the remaining 1,310 studies were screened. Of these, 1,270 studies were excluded due to irrelevance. The full texts of the remaining 40 studies, along with 12 additional studies identified through reference lists of relevant systematic reviews, were assessed for eligibility. A total of 31 studies were excluded for the following reasons: 4 studies were unavailable in full text, 11 studies did not report the outcomes of interest or had incomplete results, 9 studies were not RCTs, 3 studies included ineligible populations, 2 studies had ineligible comparators, 1 study involved concurrent first-line treatment for endometriosis that had a significant impact on the target outcomes, and 1 study was not published in English. Ultimately, 21 studies were included in the systematic review and meta-analysis.

### 3.2 Study characteristics

The characteristics of the included studies are summarized in Supplementary Table S1 [ID:1 (20), ID:2 (28), ID:3 (17), ID:4 (21), ID:5 (22), ID:6 (29), ID:7 (30), ID:8 (31), ID:9 (32), ID:10 (33), ID:11 (34), ID:12 (19), ID:13 (35), ID:14 (18), ID:15 (36), ID:16 (37), ID:17 (38), ID:18 (39), ID:19 (40), ID:20 (41), ID:21 (42)].

#### 3.2.1 Basic information

These 21 studies were published between 1997 and 2025 and originated from nine countries. Seven studies were conducted in Iran (ID: 1, 3, 5, 10, 15, 17, 19), three in the United States (ID: 7, 8, 18), two in Egypt (ID: 2, 9), two in Mexico (ID: 6, 20), two in Spain (ID: 11, 12), and two in Italy (ID: 16, 21). One study each was conducted in China (ID: 4), Canada (ID: 13), and Brazil (ID: 14). All studies were clinical RCTs.

#### 3.2.2 Study population

A total of 1,626 women diagnosed with endometriosis were included in the analysis, with 818 women receiving antioxidant supplementation as the intervention group and 808 women receiving placebo or no treatment as the control group. The mean age of the women ranged from 18.9 years (ID: 7) to 38.03 years (ID: 4). Seventeen studies diagnosed endometriosis through laparoscopy or laparotomy (ID: 1, 3, 4, 6–8, 10–18, 20, 21), two studies used ultrasound for diagnosis (ID: 9, 19), and two studies did not clearly report the diagnostic methods (ID: 2, 5). Eight studies included patients with mixed-stage endometriosis (stages I, II, III, IV) (ID: 1, 3, 4, 7, 8, 10, 13, 14), five studies included only patients with minimal to mild endometriosis (stages I-II) (ID: 6, 11, 12, 16, 20), and four studies included only patients with moderate to severe endometriosis (stages III-IV) (ID: 15, 17, 19, 21). The remaining four studies did not report disease staging (ID: 2, 5, 9, 18).

#### 3.2.3 Interventions

The included studies covered over 10 types of antioxidant agents. Nine studies used vitamins as the antioxidant intervention. Among them, one study administered vitamin C (1,000 mg/day) (ID: 4); three studies used vitamin D at doses of 50,000 IU/week (ID: 1, 5) or 2,000 IU/day (ID: 7); the remaining five studies used a combination of vitamin C and vitamin E (with vitamin C doses of 343 mg/day or 1,000 mg/day, and vitamin E doses of 800 IU/day, 1,200 IU/day, or 84 mg/day) (ID: 2, 3, 6, 8, 9). Three studies used pentoxifylline (800 mg/day) as the antioxidant agent (ID: 10–12). Two studies used melatonin at doses of 5 mg/day or 10 mg/day (ID: 13, 14). One study each used the following antioxidant agents: fish oil (1,000 mg/day) (ID: 7), astaxanthin (6 mg/day) (ID: 15), N-palmitoylethanolamine (800 mg/day) combined with transpolydatin (80 mg/day) (ID: 16), garlic tablets (providing 1,100 μg allicin/day) (ID: 17), resveratrol (40 mg/day) (ID: 18), and silymarin (280 mg/day) (ID: 19). In addition, two studies used a combination of more than three antioxidant agents as the intervention (ID: 20, 21).

Eight studies had an intervention duration of 2 months or less (ID: 2–4, 8, 9, 13, 14, 18), seven studies lasted 3 months (ID: 1, 5, 11, 15–17, 19), one study lasted 4 months (ID: 20), and five studies lasted 6 months (ID: 6, 7, 10, 12, 21).

Four studies allowed all participants to use analgesics as needed. Among them, three studies (ID: 7, 14, 18) explicitly reported that the use of analgesics did not significantly interfere with the evaluation of the therapeutic effect of antioxidants. However, one study (ID: 13) did not quantify the difference in analgesic use between the two groups nor include it as a covariate in the statistical model, thus the potential confounding effect of analgesics on treatment efficacy could not be fully excluded.

A total of four studies involved concurrent first-line hormonal therapy for endometriosis. In the study by Nodler et al. (ID: 7), most participants (>90%) initiated hormonal therapy (combination hormones, progestin-only hormones, or leuprolide acetate) at baseline, and sensitivity analyses showed no significant interference with antioxidant efficacy. In the study by Amirsalari et al. (ID: 17), all participants received uniform standard hormonal therapy, and despite the lack of separate confounding analysis, consistent baseline treatment and ANCOVA adjustment supported minimal interference. Mendes da Silva et al. (ID: 18) reported that all participants used the same combined oral contraceptive (0.15 mg levonorgestrel + 0.03 mg ethinyl estradiol), which served as a consistent background treatment and did not affect the assessment of resveratrol. In the study by Mirzaei et al. (ID: 19), all participants took the same dose of dienogest, and while the independent effect of silymarin could not be isolated, uniform hormonal therapy supported the reliability of efficacy evaluation.

### 3.3 Risk of bias

**Figure 2:**
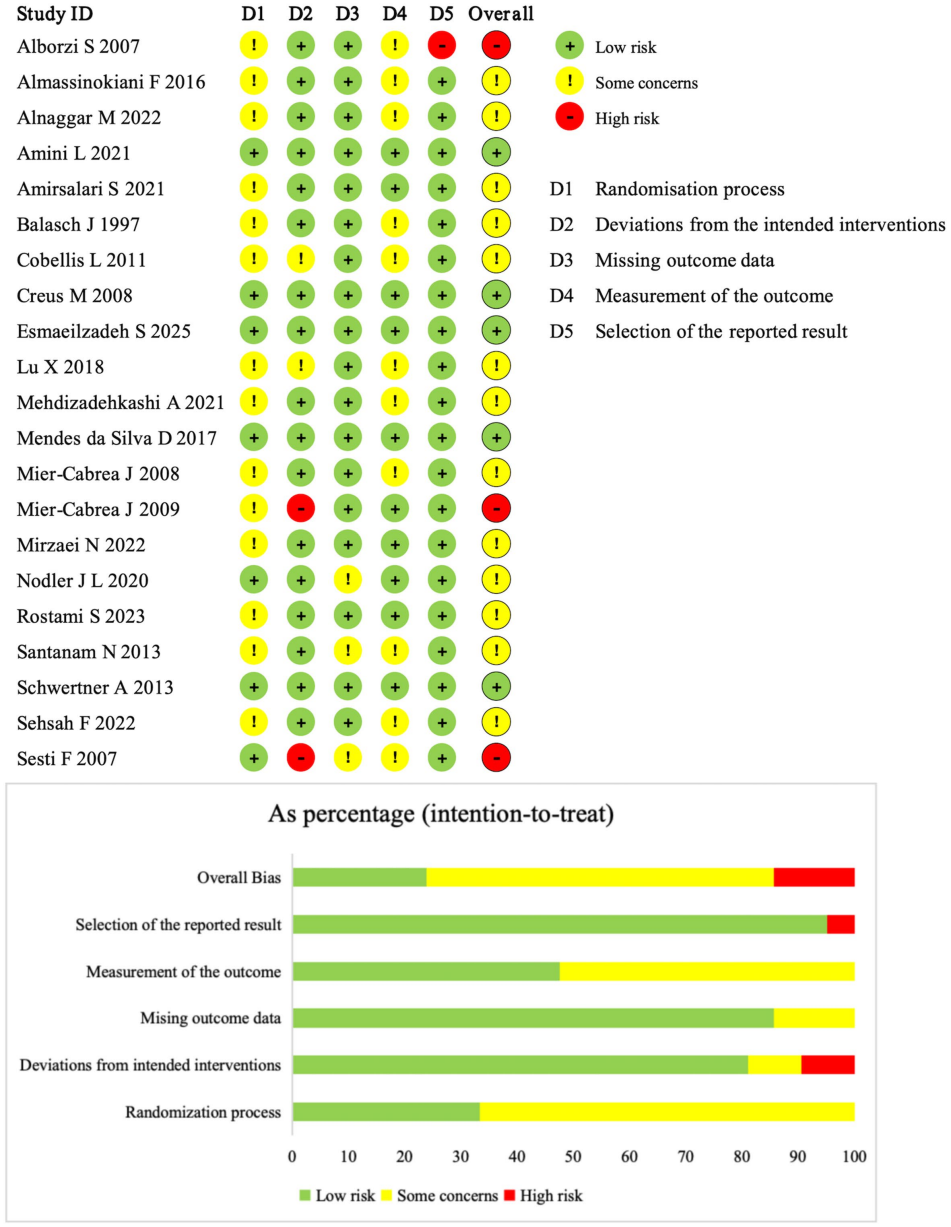
Risk of bias assessment for included studies.

Figure 2 summarizes the risk of bias of the included studies. Five studies (ID: 3, 12, 13, 14, 18) were judged as low risk of bias across all domains. Two studies (ID: 20, 21) were rated as high risk of bias in the domain of deviations from the intended interventions, because the interventions involved dietary antioxidant therapy and neither participants nor study personnel were blinded. One study (ID: 10) was judged as high risk of bias in the domain of selection of the reported result, due to inconsistency in the reported intervention duration between the abstract and the main text. The remaining studies were rated as having some concerns of risk of bias, most commonly due to insufficient details on the randomization process, blinding procedures, or missing outcome data.

### 3.4 Primary outcomes

#### 3.4.1 Pelvic pain

**Figure 3:**
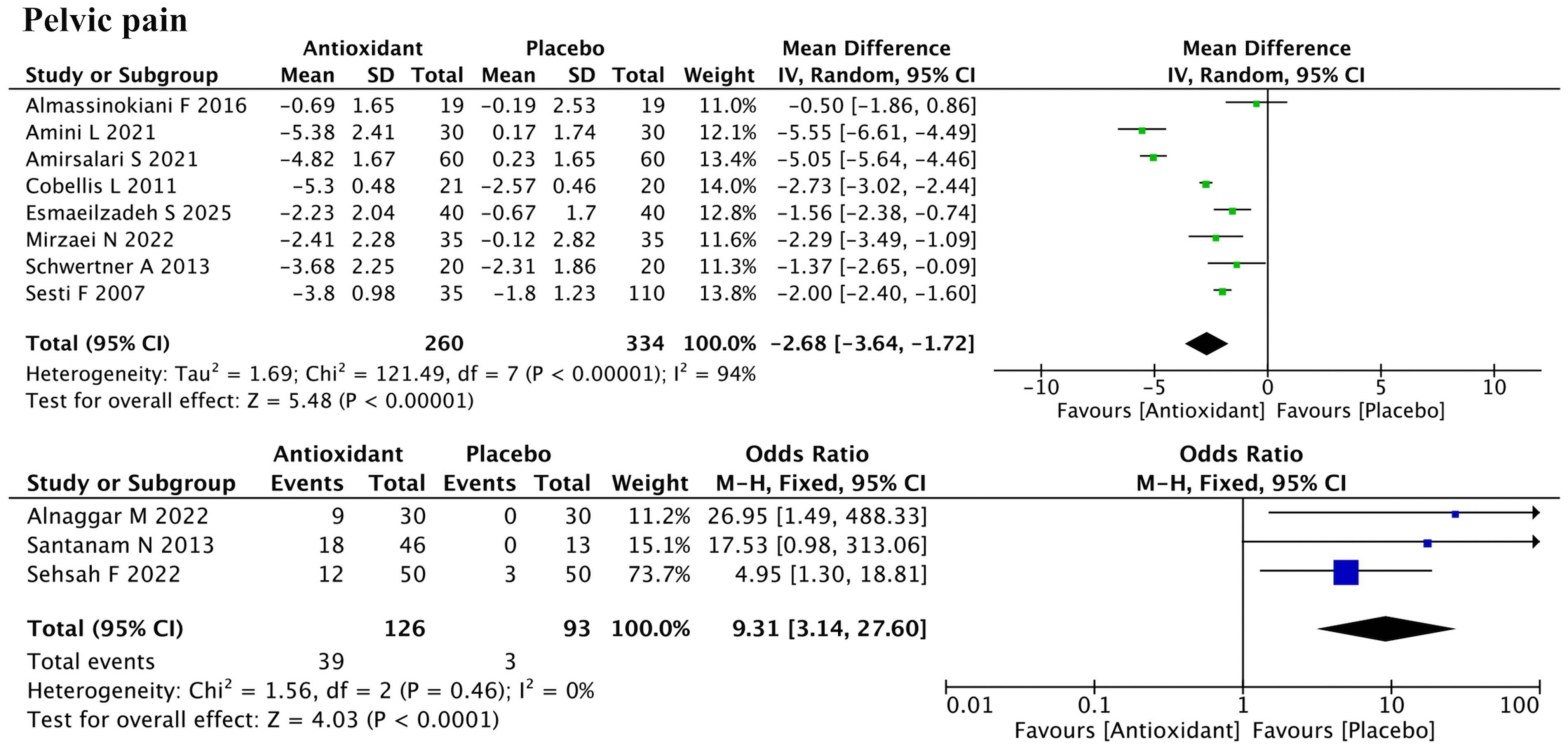
Forest plot of the effect of antioxidant supplementation on pelvic pain.

Eleven studies assessed pelvic pain, including 8 using continuous outcomes (change in Visual Analog Scale (VAS) scores) and 3 using dichotomous outcomes (pain relief: yes/no) (Figure 3). Meta-analysis of the continuous data indicated a significant reduction in pelvic pain (SMD = −2.68; 95% CI: −3.64, −1.72; *p* < 0.00001; *I*^2^ = 94%, random-effects model). The three binary studies also supported efficacy (RR = 9.31; 95% CI: 3.14, 27.60; *p* < 0.0001; *I*^2^ = 0%, fixed-effect model). Subgroup analysis revealed that the antioxidant type significantly influenced the treatment effect (P for interaction < 0.00001), constituting a major source of heterogeneity (Supplementary Figure S1a). Vitamins showed no significant effect (SMD = −3.04; *p* = 0.23), while combined antioxidants (SMD = −2.38; *p* < 0.00001) and melatonin (SMD = −1.50; *p* < 0.0001) showed significant benefits. Garlic tablet (SMD = −5.05) and silymarin (SMD = −2.29) also showed apparent effects in single studies, but no firm conclusions can be drawn due to limited data. Sensitivity analysis confirmed the robustness of results (Supplementary Figure S2a).

#### 3.4.2 Dysmenorrhea

**Figure 4:**
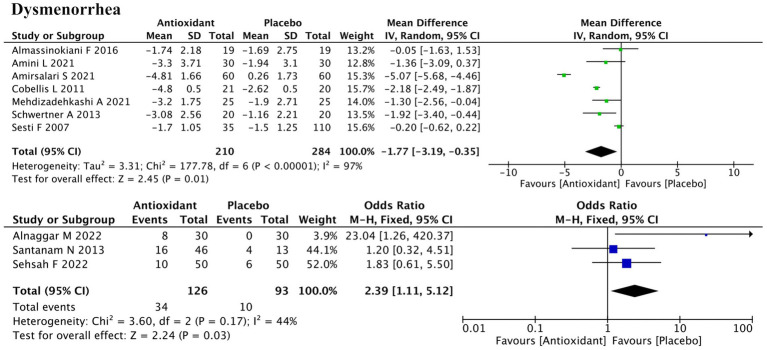
Forest plot of the effect of antioxidant supplementation on dysmenorrhea.

Ten studies evaluated dysmenorrhea, with 7 using continuous data and 3 using dichotomous outcomes (Figure 4). Meta-analysis showed significant improvement in both (SMD = −1.77; 95% CI: −3.19, −0.35; *p* = 0.01; *I*^2^ = 97%, random-effects model; and RR = 2.39; 95% CI: 1.11, 5.12; *p* = 0.03; *I*^2^ = 44%, fixed-effect model). Subgroup analysis revealed that antioxidant type, disease stage, and concurrent hormonal therapy were the major sources of heterogeneity (all P for interaction < 0.00001) (Supplementary Figure S1b). Among antioxidant types, the effect of combined antioxidant supplementation was not statistically significant (SMD = −1.20; *p* = 0.23), whereas vitamins demonstrated significant improvement (SMD = −0.95; *p* = 0.03). Significant improvement was also observed in the subgroup without concurrent hormonal therapy (SMD = −1.18; *p* = 0.03) and in the mixed-stage endometriosis subgroup (SMD = −4.92; *p* < 0.00001). Some subgroups (e.g., melatonin, stage I/II disease) contained only one study, and thus were not further elaborated due to insufficient evidence for reliable conclusions. Sensitivity analysis showed that excluding the study by Cobellis et al. (ID: 16) changed the *p*-value from 0.01 to 0.13, suggesting a substantial influence on the overall result. Heterogeneity remained high after excluding any individual study, indicating that it likely stemmed from systematic differences rather than any single source (Supplementary Figure S2b).

#### 3.4.3 Dyspareunia

**Figure 5:**
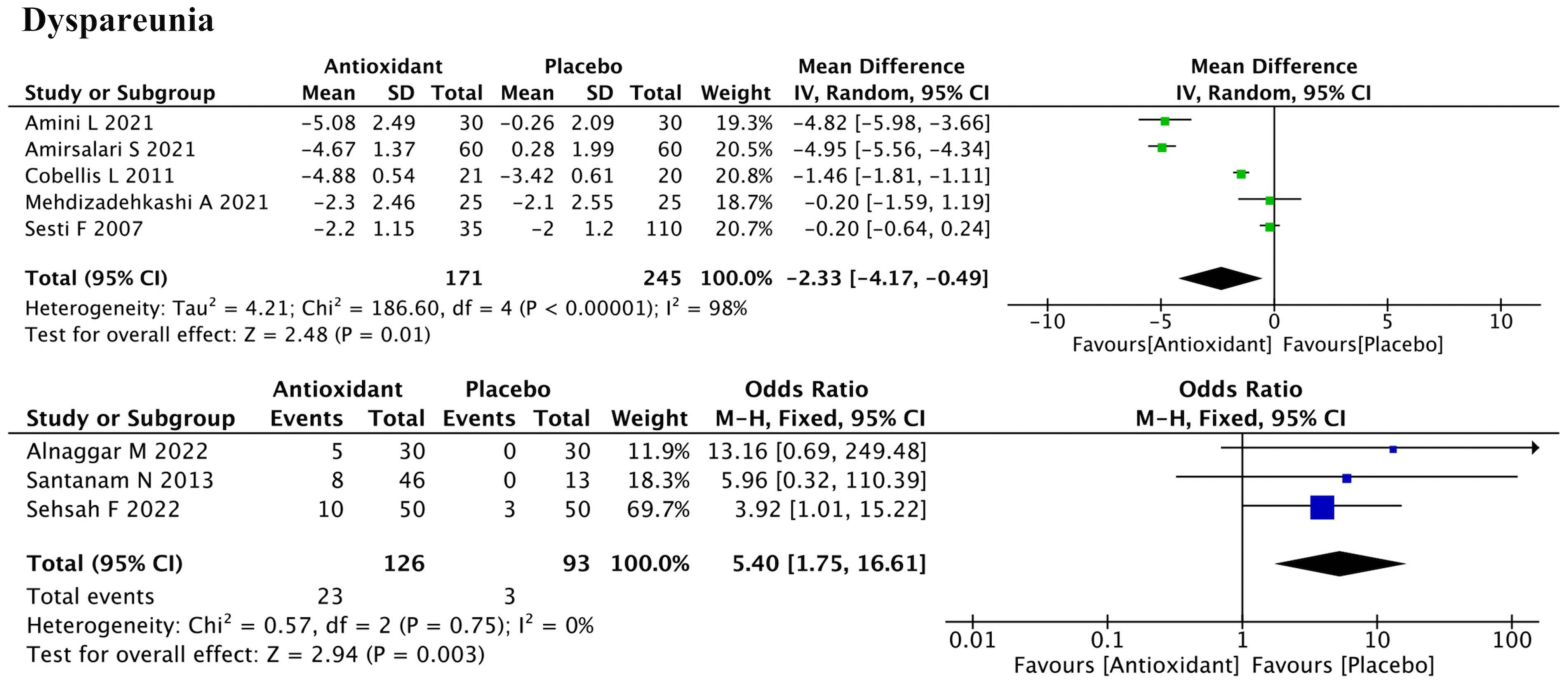
Forest plot of the effect of antioxidant supplementation on dyspareunia.

Dyspareunia outcomes were reported in eight studies—five using continuous data and three using dichotomous outcomes (Figure 5). Meta-analysis demonstrated significant improvement with antioxidant supplementation for both outcome types (SMD = −2.33; 95% CI: −4.17, −0.49; *p* = 0.01; *I*^2^ = 98%, random-effects model; and RR = 5.40; 95% CI: 1.75, 16.61; *p* = 0.003; *I*^2^ = 0%, fixed-effect model). Subgroup analysis indicated that antioxidant type, concurrent hormonal therapy, intervention duration, and disease stage were potential sources of heterogeneity (all P for interaction < 0.00001) (Supplementary Figure S1c). Among antioxidant types, neither vitamins (SMD = −2.53; *p* = 0.27) nor combined antioxidants (SMD = −0.84; *p* = 0.18) showed statistically significant effects. Additionally, significant improvement was observed in the subgroup without hormonal therapy (SMD = −1.63; *p* = 0.02), in patients with mixed-stage disease (SMD = −4.92; *p* < 0.00001), while the 3-month intervention subgroup did not show significant improvement (SMD = −2.25; *p* = 0.10). However, some subgroups based on a single study were not further discussed, as definitive conclusions could not be drawn. Sensitivity analysis showed that excluding either the study by Amini L (ID: 3) or Cobellis L (ID: 16) increased the *p*-value from 0.01 to 0.09, suggesting notable influence on the pooled result. Heterogeneity remained high (*I*^2^ > 90%) regardless of which study was removed, indicating that it likely originated from systematic differences rather than the effect of any single study (Supplementary Figure S2c).

#### 3.4.4 Overall pain

**Figure 6:**
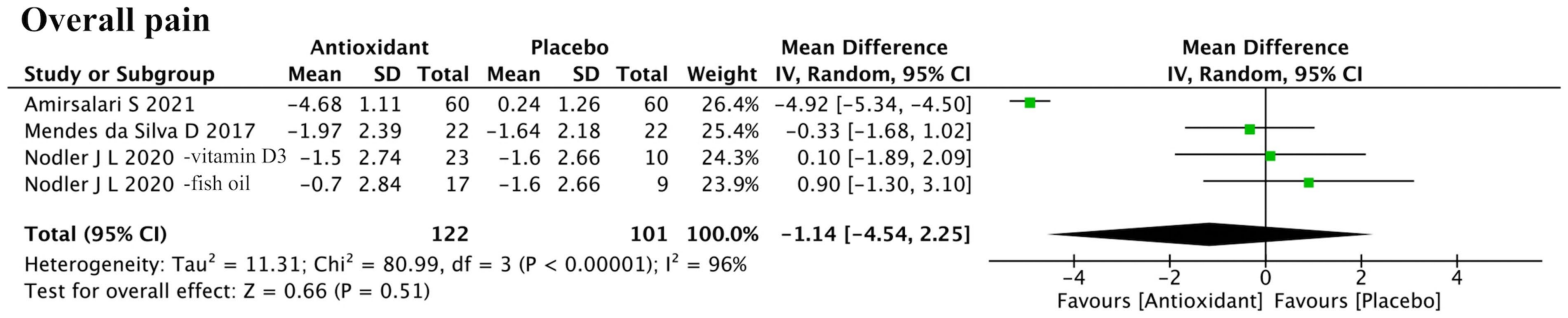
Forest plot of the effect of antioxidant supplementation on overall pain.

Four studies assessed overall pain (Figure 6). Meta-analysis found no significant difference between antioxidant and control groups (SMD = −1.14; 95% CI: −4.54, 2.25; *p* = 0.51; *I*^2^ = 96%, random-effects model). Sensitivity analysis identified Amirsalari et al. (ID: 17) as the main source of heterogeneity. After its exclusion, heterogeneity dropped (*I*^2^ = 0%), but the pooled result remained nonsignificant (SMD = 0.03; 95% CI: −0.97, 1.03; *p* = 0.95, fixed-effect model) (Supplementary Figure S2d).

### 3.5 Secondary outcomes

#### 3.5.1 Oxidative stress biomarkers

**Figure 7:**
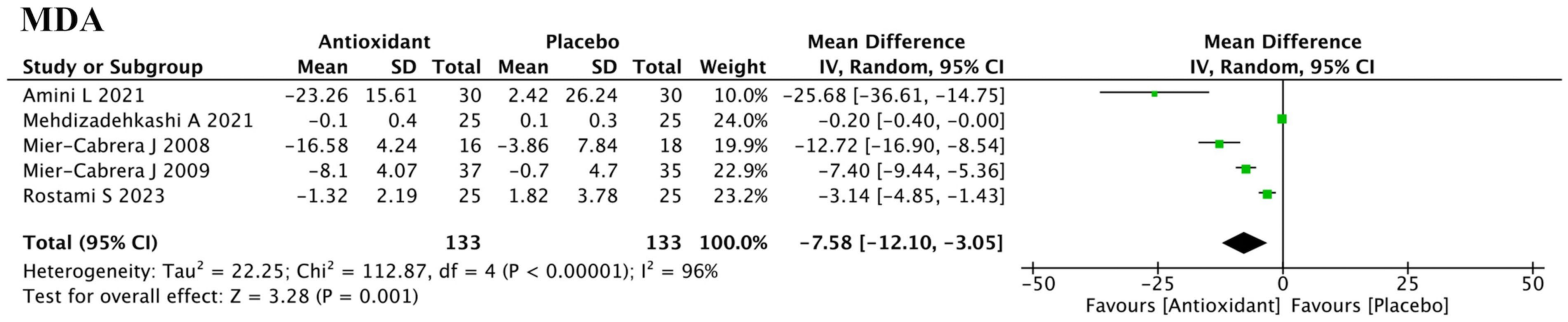
Forest plot of the effect of antioxidant supplementation on MDA.

Eight studies reported changes in oxidative stress-related biomarkers (Figure 7). Meta-analysis of 5 studies assessing malondialdehyde (MDA) levels in peripheral blood showed a significant reduction with antioxidant supplementation (SMD = −7.58; 95% CI: −12.10, −3.05; *p* = 0.001; *I*^2^ = 96%, random-effects model). Subgroup analysis identified antioxidant type as a major contributor to heterogeneity (P for interaction = 0.004) (Supplementary Figure S1d). Vitamin-based interventions showed a large but nonsignificant effect (SMD = −11.80; *p* = 0.06), likely due to high heterogeneity (*I*^2^ = 96%). Sensitivity analysis confirmed the robustness of the results (Supplementary Figure S2e). Three studies (ID: 3, 5, 15) assessed total antioxidant capacity (TAC), but one (ID: 3) reported implausibly low values and was excluded from quantitative synthesis. No meta-analysis was conducted for TAC, superoxide dismutase (SOD), or lipid hydroperoxide (LOOH) due to insufficient data. Descriptive analysis showed that vitamin D, astaxanthin and high-antioxidant diets increased TAC and SOD levels (ID: 5, 15, 20), while vitamin C/E supplementation and antioxidant diets reduced LOOH concentrations (ID: 6, 20).

#### 3.5.2 Clinical pregnancy rate

**Figure 8:**
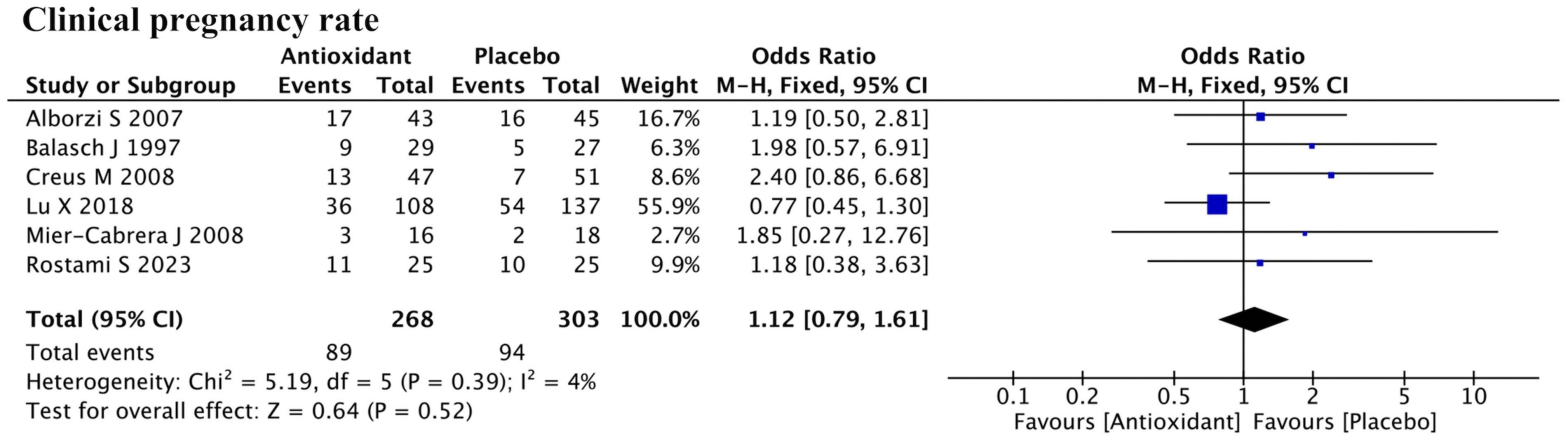
Forest plot of the effect of antioxidant supplementation on clinical pregnancy rate.

A total of six studies reported clinical pregnancy rate (Figure 8). Meta-analysis indicated that antioxidant supplementation did not significantly improve the clinical pregnancy rate in patients with endometriosis compared to the control group (RR = 1.12; 95% CI: 0.79, 1.61; *p* = 0.52; *I*^2^ = 4%, fixed-effect model). Further subgroup analyses showed that neither the use of assisted reproductive technology nor the type of antioxidant had a significant impact on the overall outcome (Supplementary Figure S1e).

### 3.6 Quality of evidence

According to the GRADE assessment, the certainty of evidence for pelvic pain and dyspareunia based on dichotomous outcomes was rated as moderate, while the certainty of evidence for all other outcomes was rated as low or very low (Supplementary Figure S3). The main reasons for downgrading included risk of bias (*n* = 9), publication bias (*n* = 9), inconsistency of results (*n* = 4), and imprecision (*n* = 2). At the same time, several outcomes were upgraded due to a large magnitude of effect (*n* = 5).

### 3.7 Safety and adverse events

Six of the included studies reported safety outcomes related to antioxidant supplementation. Five studies (ID: 13, 14, 15, 16, 19) explicitly stated that no adverse events occurred. One study (ID: 18), in which all participants received combined oral contraceptives (COCs) as background therapy, reported adverse events associated with concomitant use of COCs and resveratrol, including headache (*n* = 6), diplopia (*n* = 1), and reduced libido (*n* = 1). The remaining studies did not explicitly report safety outcomes; however, no withdrawals due to intolerance were documented in their study flow diagrams.

## 4 Discussion

This systematic review and meta-analysis included 21 RCTs involving 1,626 women with endometriosis, evaluating the effects of over 10 antioxidant types on pain, oxidative stress biomarkers, and clinical pregnancy outcomes. The results showed that antioxidant supplementation significantly relieved certain types of endometriosis-associated pain—specifically pelvic pain, dysmenorrhea, and dyspareunia—and reduced peripheral blood levels of MDA. However, no significant improvement was found for overall pain or clinical pregnancy rate. Heterogeneity was evident and appeared to be influenced by antioxidant type, disease stage, and intervention duration. It is worth emphasizing that, given the complex pathophysiology and heterogeneous clinical manifestations of endometriosis, while antioxidants demonstrate significant effects in some areas, their overall impact remains relatively limited and they should be considered as adjunctive therapies rather than replacements for standard surgical or hormonal treatments.

Pain is one of the most common clinical symptom in endometriosis, typically manifesting as chronic pelvic pain, dysmenorrhea or dyspareunia (9, 43). Although existing treatments including analgesics, hormonal therapy, and laparoscopy are commonly used, they face challenges like side effects, contraindications in pregnancy, and high recurrence (44). Oxidative stress plays a key role in pain pathophysiology by activating macrophages and releasing inflammatory mediators (e.g., TNF-α, IL-6, PGE_2_), promoting neurogenic inflammation, and inducing lipid peroxidation products that stimulate nociceptors (9, 45, 46). It also activates NF-κB and COX-2 pathways, forming a vicious cycle of oxidative stress and pain (47).

Several *in vitro* and *in vivo* studies have explored the therapeutic effects and mechanisms of antioxidants in endometriosis. Vitamin C significantly reduced the size of endometriotic cysts in rats (48). Melatonin inhibited the endometrial epithelial cell proliferation and suppress both lipid and protein oxidation (49, 50). Curcumin downregulated angiogenic and pro-inflammatory factors via NF-κB pathway inhibition, reducing ectopic lesion growth in animal models (51, 52). These findings suggest that antioxidant therapies targeting free radical scavenging and immune modulation may represent promising strategies for relieving endometriosis-associated pain. Consistent with these mechanisms, our meta-analysis demonstrated that antioxidants significantly improved specific types of pain such as pelvic pain, dysmenorrhea, and dyspareunia. However, no benefit was observed for overall pain likely due to heterogeneity in the definition of “overall pain,” which in the included studies often referred to non-specific terms such as “worst pain,” “endometriosis-associated pain,” or simply “pain.” Although all were assessed using the VAS, variations in timing, location, and interpretation could have introduced inconsistency. Additionally, overall pain often represents a composite score encompassing multiple pain types or anatomical regions, potentially masking improvements in specific symptoms and thereby reducing statistical power. Future studies should use standardized, clearly defined pain outcomes to improve comparability and sensitivity.

Oxidative stress biomarkers reflect the body’s redox balance, including markers of damage and antioxidant defense (53). Abnormal levels have been observed in the blood, peritoneal fluid, and follicular fluid of endometriosis patients, indicating a pronounced oxidative stress state (54–57). Our analysis showed that antioxidants significantly reduced MDA, a terminal product of lipid peroxidation, indicating that antioxidants may mitigate disease progression by inhibiting lipid oxidative damage. This aligns with prior findings that vitamins, melatonin, and other antioxidants reduce MDA in various conditions (58–61). Vitamin E can donate electrons to lipid peroxyl radicals, thereby interrupting lipid peroxidation chain reactions, while melatonin directly neutralizes ROS and reactive nitrogen species through cascade reaction mechanisms, generating antioxidant metabolites and efficiently scavenging free radicals (58, 62). In addition to MDA, TAC and SOD, which reflect antioxidant defense, and LOOH, an indicator of lipid oxidative injury, were also analyzed descriptively (53, 63). Although meta-analyses were not performed due to limited study numbers or heterogeneity, available evidence suggests that antioxidants may increase TAC and SOD while reducing LOOH, supporting their potential role in attenuating oxidative damage. Further standardized RCTs are needed to confirm these trends and their clinical relevance.

Endometriosis is a major cause of infertility in women of reproductive age, with approximately one-third of patients affected by infertility (64). Despite benefits on pain and oxidative stress, our results showed no significant effect of antioxidants on clinical pregnancy rates. This may relate to the multifactorial etiology of endometriosis-related infertility, involving anatomical abnormalities, impaired oocyte quality, dysregulated immune microenvironment, and reduced endometrial receptivity (65). Oxidative stress has been shown to disrupt oocyte maturation, embryo implantation, and corpus luteum maintenance, thereby compromising fertility potential in women with endometriosis (66). Some *in vitro* and animal studies have also demonstrated that antioxidants may enhance endometrial decidualization and improve pregnancy outcomes (67, 68). In addition, one cohort study reported that after 3 months of N-acetylcysteine (NAC) treatment, 39 out of 52 endometriosis patients (75%) with fertility desire conceived naturally, and an additional 6 (11.5%) achieved pregnancy through assisted reproductive technologies (ART), suggesting a possible benefit of NAC in enhancing reproductive outcomes. However, current RCTs have not provided robust clinical evidence to support a definitive fertility-enhancing effect of antioxidants in this population (69). Overall, existing clinical data remain insufficient to confirm the therapeutic efficacy of antioxidants in improving fertility in women with endometriosis, and further high-quality research is needed.

Our subgroup analyses provided insights into factors influencing treatment efficacy. Vitamin-based interventions showed varying effects across pain types. Continuous outcomes indicated significant improvement in dysmenorrhea only, while three studies using dichotomous outcomes found consistent benefits of combined vitamin C and E supplementation across pelvic pain, dysmenorrhea, and dyspareunia. This suggests that multivitamin combinations may be more effective than single-agent regimens. In terms of disease stage, antioxidant treatment significantly improved pelvic pain, dysmenorrhea, and dyspareunia in patients with minimal/mild (stage I/II) endometriosis, but showed significant improvement only for pelvic pain in moderate/severe (stage III/IV) patients. In mixed-stage populations, significant improvements were observed for pelvic pain and dyspareunia, whereas dysmenorrhea did not show a significant benefit. These findings suggest that patients with minimal/mild endometriosis may derive greater benefit in terms of pain management from antioxidant adjunctive therapy, while disease progression in moderate/severe patients may limit treatment effects. The opposing effects within mixed-stage groups may have masked the true therapeutic benefit; further studies are needed to clarify differential responses across disease stages. Regarding intervention duration, 3-month treatments significantly improved pelvic pain and dysmenorrhea, but did not significantly alleviate dyspareunia. This may indicate that dyspareunia requires a longer treatment duration for observable benefits. However, since only one study with a 6-month intervention reported pain outcomes, the optimal duration for relieving dyspareunia remains uncertain and requires further investigation to determine pain type-specific treatment timelines.

Previous meta-analyses have predominantly focused on specific antioxidants or single outcomes. Shrateh et al. (23) included three RCTs and found that vitamin D relieved dysmenorrhea and dyspareunia but not pelvic pain in endometriosis women—partly consistent with our findings. Bayu et al. (24) analyzed five RCTs of combined vitamin C and E for endometriosis patients, reporting significant improvements in chronic pelvic pain, dysmenorrhea, and dyspareunia, aligning with our results. Zheng et al. (70) focused on vitamin-based antioxidants and found that vitamin could alleviate endometriosis-associated pain and reduce plasma MDA levels, but limited data precluded the meta-analysis on fertility outcomes. Additionally, Baradwan et al. (25) reviewed 10 RCTs on endometriosis-related pain and found benefits for dysmenorrhea and pelvic pain, but not dyspareunia. However, inclusion of a study on primary dysmenorrhea, rather than in women with endometriosis, likely introduced bias and reduced the reliability of their findings. In contrast, our study applied stricter inclusion criteria, included more RCTs, examined a wider range of antioxidants, and assessed pain alongside oxidative stress and fertility.

This study has several strengths. It is the first review to comprehensively assess over 10 antioxidant agents for three key outcomes in endometriosis: pain, oxidative stress, and fertility. Subgroup analyses further examined factors that may affect efficacy, including antioxidant type, treatment duration, and disease stage. However, several limitations should be noted. Although all included studies were RCTs, many had small samples and showed methodological differences in diagnosis, interventions, and outcome definitions. Due to the limited number of studies per outcome, funnel plots were not generated, and formal assessment of publication bias could not be performed. Moreover, short follow-up durations limited evaluation of long-term outcomes such as fertility. Future research should focus on large, multicenter, standardized RCTs with longer follow-up periods to better assess the efficacy and safety of antioxidant interventions and to inform clinical practice.

## 5 Conclusion

This systematic review and meta-analysis suggests that antioxidant supplementation may have beneficial effects in alleviating specific types of endometriosis-associated pain—including pelvic pain, dysmenorrhea, and dyspareunia—and in reducing oxidative stress levels, particularly peripheral MDA. However, no significant improvement was observed in overall pain or clinical pregnancy rate. Subgroup analyses indicate that treatment efficacy may vary depending on the type of antioxidant, disease stage, and duration of intervention. These findings support the potential role of antioxidant supplementation as the adjunctive therapy for managing pain and oxidative stress in endometriosis. Despite these promising results, current evidence is limited by methodological heterogeneity and small sample sizes. Future large-scale and high-quality RCTs are warranted to validate these findings, identify the most effective antioxidant regimens, and assess their long-term effects on fertility and disease recurrence.

## Data Availability

The original contributions presented in the study are included in the article/Supplementary material, further inquiries can be directed to the corresponding author.

## Footnotes

^1^ https://www.crd.york.ac.uk/prospero/display_record.php?ID=CRD420251071723

^2^ https://gdt.gradepro.org/

## Supplementary material

SUPPLEMENTARY FIGURE 1 Summary of subgroup analyses for various outcomes.

SUPPLEMENTARY FIGURE 2 Sensitivity analysis of included trials for various outcomes.

SUPPLEMENTARY FIGURE 3 GRADE evidence profiles.

SUPPLEMENTARY TABLE 1 Characteristics of included studies.
