# Supplementary figures and images for: The effects of antioxidant supplementation on pain, oxidative stress markers, and clinical pregnancy rate in women with endometriosis: a systematic review and meta-analysis of randomized controlled trials
Source: Front Med (Lausanne). 2025 Oct 29;12:1694281. doi: 10.3389/fmed.2025.1694281 (PMC12605171; doi:10.3389/fmed.2025.1694281)

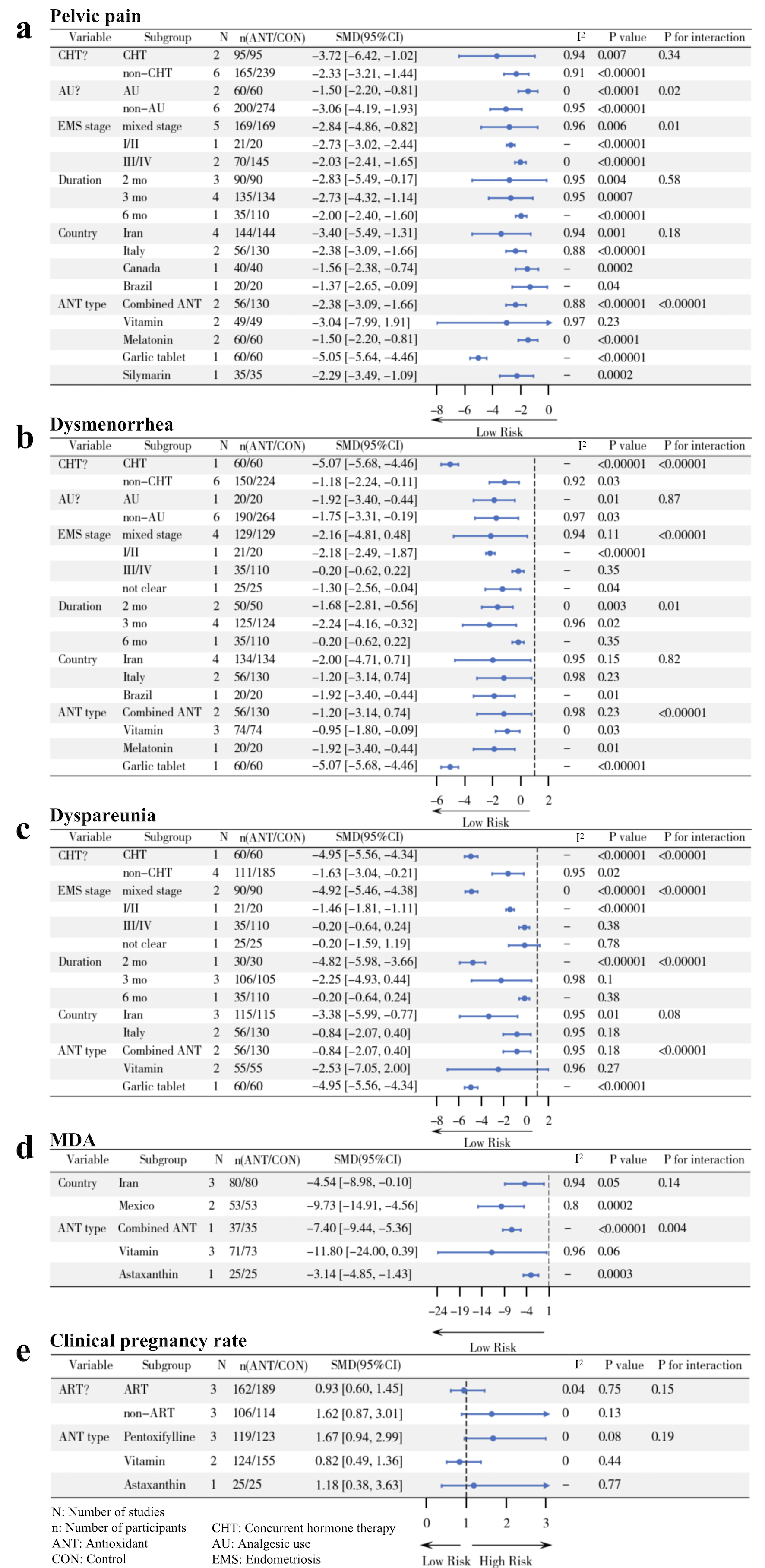

Supplement: SUPPLEMENTARY FIGURE 1 — Summary of subgroup analyses for various outcomes. [file Image_1.tif]

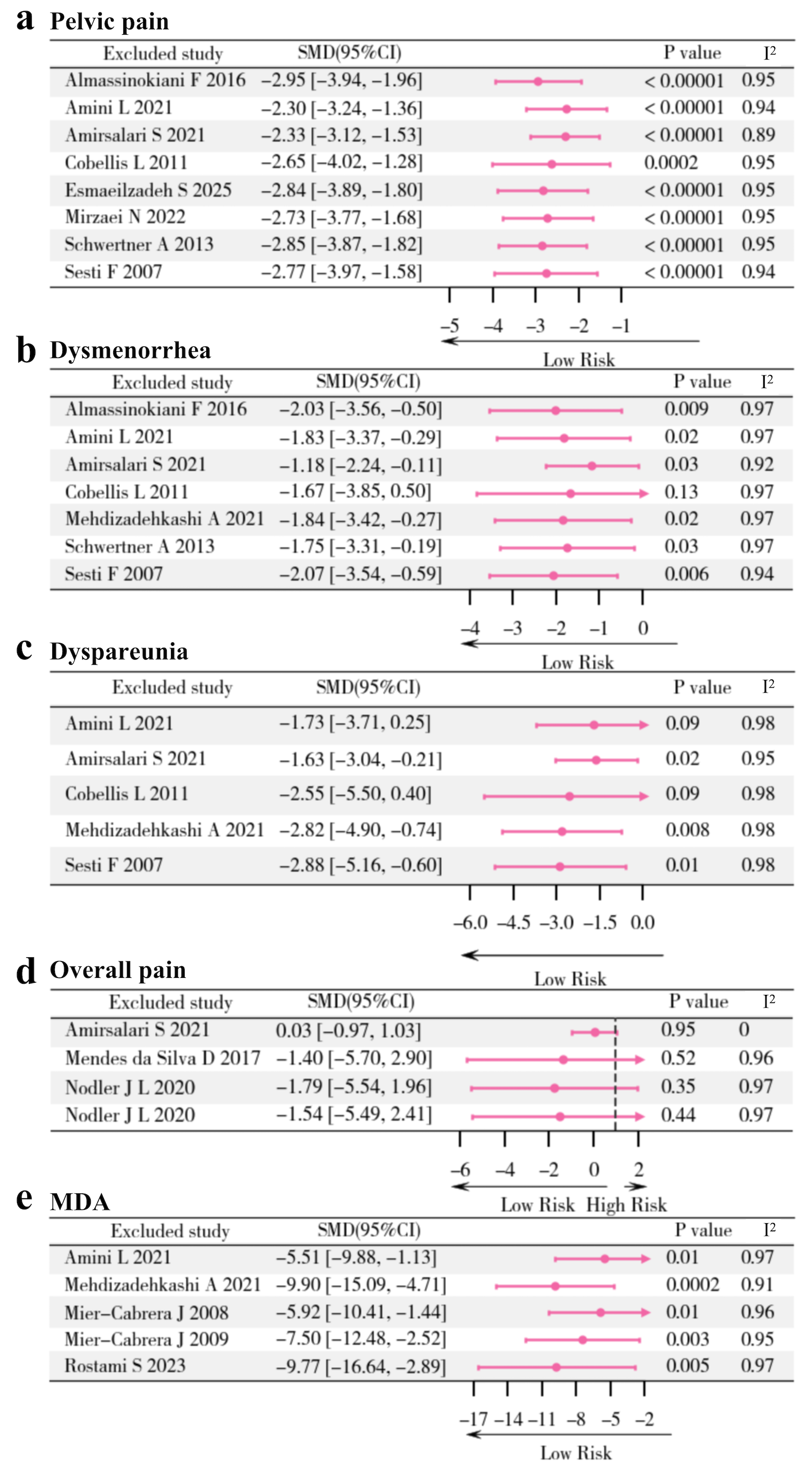

Supplement: SUPPLEMENTARY FIGURE 2 — Sensitivity analysis of included trials for various outcomes. [file Image_2.tif]

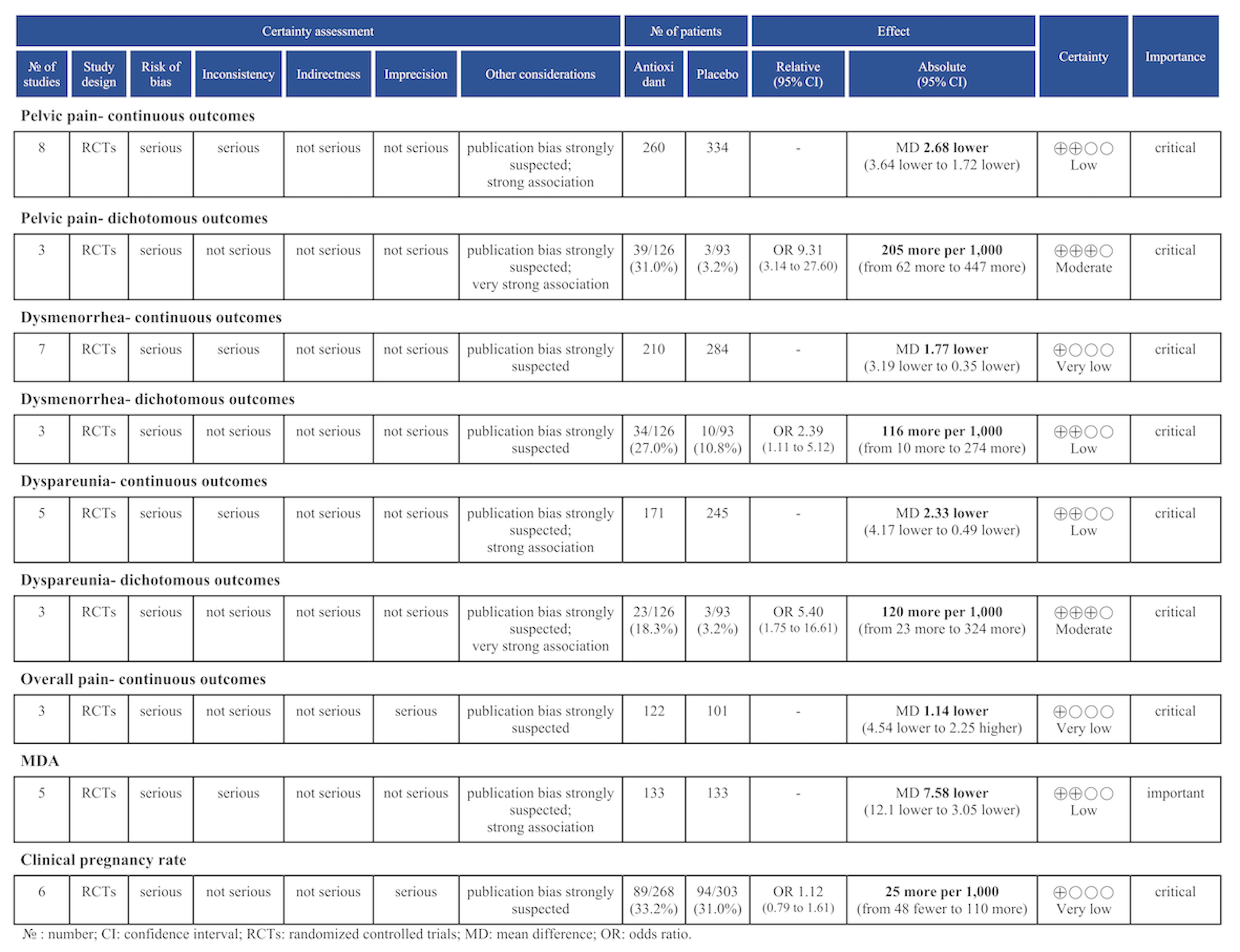

Supplement: SUPPLEMENTARY FIGURE 3 — GRADE evidence profiles. [file Image_3.tif]
